# Lifelong vitamin B_12_ monitoring after gastrectomy: A case report of subacute combined degeneration with 8-year latency in an alcoholic

**DOI:** 10.1097/MD.0000000000048097

**Published:** 2026-03-20

**Authors:** Hee Kyung Cho, Kang Lip Kim

**Affiliations:** a Department of Physical Medicine and Rehabilitation, Catholic University of Daegu School of Medicine, Daegu, Republic of Korea; b Department of Physical Medicine and Rehabilitation, Daegu Health College Hospital, Daegu, Republic of Korea.

**Keywords:** alcoholism, gastrectomy, neurologic manifestations, subacute combined degeneration, vitamin B_12_ deficiency

## Abstract

**Rationale:**

Although vitamin B_12_ deficiency is a well-known complication following total gastrectomy, subacute combined degeneration (SCD) rarely develops beyond 5 years postoperatively. Eight-year latency cases are rarely reported, especially when combined with chronic alcohol use, which accelerates neurologic decline and worsens prognosis. This report aims to present a rare case of delayed SCD after total gastrectomy in a patient with chronic alcoholism, emphasizing the importance of lifelong vitamin B_12_ monitoring in high-risk individuals.

**Patient concerns:**

A 49-year-old man presented with progressive quadriparesis and sensory disturbances eight years after total gastrectomy for gastric cancer. He also had a significant history of chronic alcohol consumption, raising suspicion for multifactorial vitamin B_12_ deficiency.

**Diagnoses:**

Laboratory tests revealed severe vitamin B_12_ deficiency, pancytopenia, and elevated homocysteine and methylmalonic acid levels. Cervical spine magnetic resonance imaging revealed T2-weighted hyperintensity in the dorsal columns, consistent with SCD.

**Interventions:**

The patient received vitamin B_12_ supplementation and underwent a comprehensive rehabilitation program aimed at improving motor function.

**Outcomes:**

After rehabilitation, he regained independent ambulation using a cane.

**Lessons:**

This case shows an unusually prolonged latency of SCD after total gastrectomy and is notable for the coexistence of 2 established risk factors: gastrectomy and chronic alcoholism. It emphasizes the importance of lifelong surveillance of vitamin B_12_ status to prevent avoidable and potentially irreversible neurologic complications in high-risk populations.

## 1. Introduction

Vitamin B_12_ deficiency is a recognized complication after total gastrectomy, primarily due to the loss of intrinsic factor production, which is essential for B12 absorption.^[1,2]^ While deficiency typically emerges within 1 to 3 years postoperatively, neurological sequelae such as subacute combined degeneration (SCD) typically develop after several years of uncorrected deficiency – most often within 3 to 5 years – although markedly delayed presentations up to nearly a decade have also been reported and warrant clinical attention.^[3-5]^ Chronic alcoholism increases the risk of cobalamin deficiency by reducing intake, injuring mucosa, and disrupting receptor-mediated ileal absorption.^[6,7]^ When combined with the anatomical and physiological consequences of gastrectomy, these synergistic factors may predispose patients to unusually delayed and severe neurological manifestations.

Despite these risks, postoperative follow-up in gastrectomy patients often focuses primarily on surveillance for cancer recurrence, with long-term nutritional and neurological assessments frequently overlooked.^[2,8]^ This report aims to describe a uniquely delayed case of SCD that developed after total gastrectomy in a patient with chronic alcoholism, showing the prolonged latency and the combined impact of these dual risk factors, and emphasizing the importance of lifelong vitamin B_12_ monitoring in high-risk individuals.

## 2. Case report

A 49-year-old man presented with a 5-month history of progressive weakness and sensory disturbances in both upper and lower limbs. Symptoms began with bilateral lower extremity weakness, followed by upper extremity involvement 2 months later. He had no history of trauma or prior neurological disease.

His past medical history included total gastrectomy for gastric cancer 8 years earlier, with follow-up discontinued after 1 year. He reported heavy alcohol consumption until 5 months before presentation.

Neurological examination revealed Medical Research Council grade 2 strength in the right hip flexors, grade 3 in other lower limb muscles, and grade 4 in upper limbs. Sensory examination showed decreased light touch, pinprick, and proprioception below the C5 dermatome. Bilateral ankle clonus was observed, and he was unable to stand or walk independently.

**Figure 1.**
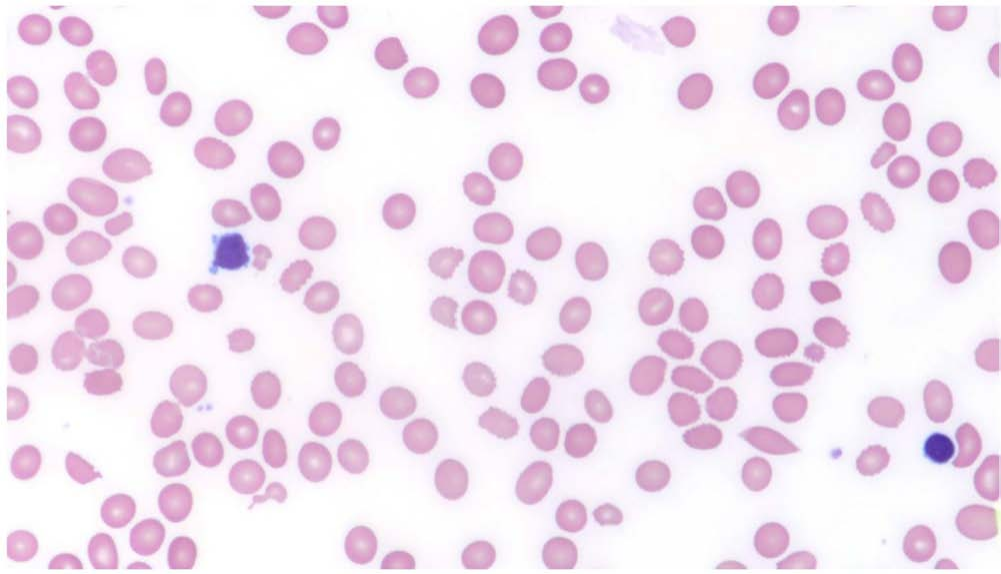
Peripheral blood smear showing macrocytic hyperchromic anemia with macro-ovalocytosis, anisocytosis, poikilocytosis, and polychromasia.

**Figure 2.**
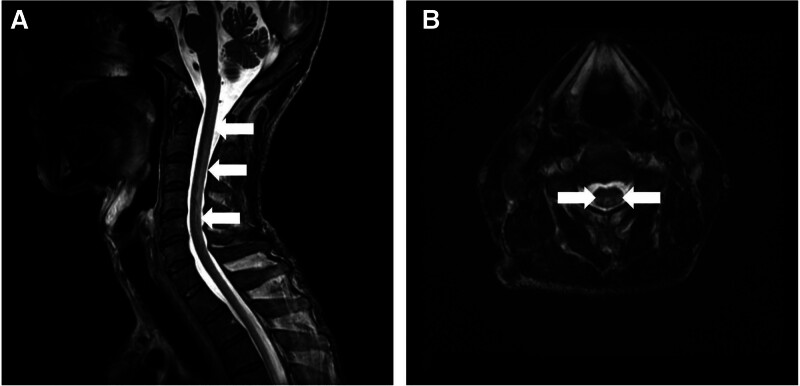
Cervical spine MRI of the patient with SCD. T2-weighted sagittal (A) and axial (B) images show high signal intensity in the dorsal columns (white arrows), consistent with demyelination. MRI = magnetic resonance imaging, SCD = subacute combined degeneration.

Laboratory investigations revealed pancytopenia, macrocytosis, low serum vitamin B_12_ (Table 1), elevated homocysteine, and methylmalonic acid levels. Peripheral blood smear revealed macrocytic hyperchromic anemia (Fig. 1). Cervical spine magnetic resonance imaging demonstrated T2 hyperintensities in the dorsal columns, consistent with SCD (Fig. 2). Somatosensory evoked potentials indicated prolonged latencies, and motor evoked potentials showed conduction delays and absent tibialis anterior responses. Nerve conduction studies revealed reduced sensory and motor amplitudes.

**Table 1:** Laboratory findings of the patient showing pancytopenia, macrocytosis, and severe vitamin B_12_ deficiency.

	Value (normal range)
WBC	3200/μL (3600–9600/μL)
Hemoglobin	8.0 g/dL (12.9–16.9 g/dL)
Platelet	88,000/μL (1,40,000–3,80,000/μL)
MCV	139.1 fL (83.7–98.5 fL)
MCH	46.1 pg (27.8–33.0 pg)
Vitamin B_12_	100 pg/mL (197–771 pg/mL)
Vitamin B1	97.6 nmol/L (66.5–220.0 nmol/L)
Homocysteine	103.1 μmol/L (<15.0 μmol/L)
Methylmalonic acid	222.4 mg/d (0.00–10.00 mg/d)
Folate	6.5 ng/mL (4.2–19.8 ng/mL)

MCH = mean corpuscular hemoglobin, MCV = mean corpuscular volume, WBC = white blood cell count.

The patient received intramuscular cobalamin 2 mg daily for 1 week, followed by 1 mg daily for another week, and thereafter 1 mg weekly until discharge. Intravenous thiamin 450 mg/d was administered for 9 days. A multidisciplinary rehabilitation program was implemented, consisting of two 30-minute physical therapy sessions and two 30-minute occupational therapy sessions daily, 5 days/wk. Physical therapy focused on progressive lower limb strengthening, balance training, proprioceptive facilitation, and gait training that progressed from standard walker-assisted ambulation to cane-assisted walking as balance and coordination improved. Since upper limb strength was relatively preserved, occupational therapy primarily focused on sensory reeducation, fine-motor coordination, and training for activities of daily living to restore functional independence. During the multidisciplinary management period, the patient was cooperative and motivated, expressing relief upon receiving a definitive diagnosis and showing optimism toward his rehabilitation outcomes.

After 6 weeks, muscle strength improved to Medical Research Council grade 4 in the lower extremities, and the patient regained independent ambulation with a monocane. A summary of key clinical milestones is presented in Table 2.^[9]^

**Table 2:** Clinical timeline of the patient.

Date	Clinical event
2011	Total gastrectomy
May 2019	Onset of neurological symptoms
October 21, 2019	Admission to neurology department
October 21–28, 2019	Diagnostic tests (biochemistry, MRI, electrophysiology)
October 23, 2019	Initiation of vitamin B_12_ supplementation
October 29, 2019	Transfer to department of rehabilitation medicine
October–November 2019	Inpatient rehabilitation
December 2019	Discharged with improved functional level

MRI = magnetic resonance imaging.

## 3. Discussion

A potentially reversible but often overlooked complication of vitamin B_12_ deficiency is SCD, which primarily involves demyelination of the dorsal and lateral columns of the spinal cord.^[1,6]^ While SCD commonly occurs within a few years of total gastrectomy due to intrinsic factor loss, our case is characterized by the coexistence of 2 established risk factors: total gastrectomy and chronic alcoholism. It is further notable for the unusually prolonged latency of 8 years and the availability of comprehensive neuroimaging, laboratory, and functional outcome data.

Gastrectomy alone profoundly disrupts vitamin B_12_ absorption due to the elimination of intrinsic factor, yet the majority of patients develop clinical deficiency within 1 to 3 years postoperatively.^[2]^ Chronic alcohol abuse independently exacerbates cobalamin deficiency through several mechanisms. First, alcohol often leads to impaired dietary intake due to poor nutritional habits and decreased appetite.^[7]^ Second, it induces direct gastrointestinal mucosal injury that interferes with the absorption of nutrients, including cobalamin.^[6,7]^ Third, alcohol impairs receptor-mediated transport, disrupting the cubilin-megalin complex in the ileum and reducing cellular uptake of vitamin B_12_.^[10]^ Finally, hepatic injury related to long-term alcohol use can compromise storage and processing of cobalamin, further accelerating deficiency.^[6,7]^

As summarized in Table 3, we reviewed recent case reports of SCD occurring after gastrectomy for comparison.^[5,11-13]^ Most patients developed neurologic manifestations within several years after surgery, although a few reports have documented longer latency periods of up to 9 years. Our patient developed SCD 8 years after subtotal gastrectomy, which falls toward the longer end of this spectrum and therefore represents an uncommon latency. Importantly, coexisting chronic alcoholism has not been reported in previous cases, making this case distinctive in showing how dual risk factors – gastrectomy and alcohol use – can synergistically exacerbate vitamin B_12_ deficiency and contribute to delayed yet severe neurologic manifestations. In contrast to previous cases without such combined risks, our patient presented with quadriparesis and marked sensory disturbance nearly a decade after gastrectomy. This was supported by biochemical evidence of cobalamin depletion, cervical magnetic resonance imaging showing dorsal column demyelination, and electrophysiological confirmation of central and peripheral conduction delays. The meaningful functional recovery achieved after timely supplementation and multidisciplinary rehabilitation further supports both the validity of the diagnosis and the importance of early recognition and intervention.^[6,14]^

**Table 3:** Summary of delayed SCD cases reported in literature, highlighting the rarity of our case in terms of latency and dual risk factors.

Case	Latency (yr)	Age	Alcohol use	B12 level	Neurological deficit	Outcome
Tsang et al^[11]^	6	35	No	Normal	Ataxic gait, sensory loss	Partial recovery
Yokoyama et al^[12]^	6	62	No	Low	Sensory loss, gait disturbance	Good recovery
Hwang et al^[5]^	9	58	No	Very low	Gait disturbance, sensory deficits	Partial recovery
Al Ani et al^[13]^	1	26	No	Low	Numbness, weakness	Partial recovery
Our case	8	49	Yes	Severely low	Quadriparesis, sensory loss	Ambulation with cane

SCD = subacute combined degeneration.

This case exposes a critical gap in current clinical practice: the lack of standardized long-term nutritional surveillance for patients after gastrectomy. In many cancer centers, follow-up efforts are rightly concentrated on oncologic recurrence during the first few years, but nutritional deficiencies with delayed manifestations – such as B12-related SCD – are often overlooked. The problem is magnified in high-risk populations such as individuals with chronic alcohol use, who are less likely to engage in routine preventive care and more likely to present with advanced disease.

Given the increasing prevalence of gastric cancer survivors globally, particularly in East Asia, the need for structured survivorship care has never been greater. Although our patient developed severe neurologic manifestations, meaningful recovery was achieved with timely recognition, supplementation, and rehabilitation. This experience shows the importance of preventing irreversible damage through proactive surveillance. Accordingly, lifelong annual vitamin B_12_ monitoring and neurological screening should be incorporated into routine postgastrectomy care.^[2]^ In high-risk groups such as individuals with chronic alcohol use, advanced age, or concomitant gastrointestinal disease, more frequent assessments at 6-month intervals may be a reasonable approach.^[6,7]^ Such tailored follow-up facilitates early detection of subclinical deficiency and allows intervention before irreversible neurologic complications develop.

## Author contributions

**Conceptualization:** Hee Kyung Cho.

**Data curation:** Hee Kyung Cho, Kang Lip Kim.

**Supervision:** Kang Lip Kim.

**Visualization:** Hee Kyung Cho, Kang Lip Kim.

**Writing – original draft:** Hee Kyung Cho, Kang Lip Kim.

**Writing – review & editing:** Hee Kyung Cho, Kang Lip Kim.
